# Performance analysis of partially shaded high-efficiency mono PERC/mono crystalline PV module under indoor and environmental conditions

**DOI:** 10.1038/s41598-024-72502-z

**Published:** 2024-09-16

**Authors:** Neha Kumari, Sanjay Kumar Singh, Sanjay Kumar, Vinay Kumar Jadoun

**Affiliations:** 1 Department of Electrical & Electronics Engineering, Amity University Rajasthan, Jaipur, India; 2 Department of Electrical Engineering, UIT, Himachal Pradesh University, Shimla, H.P. India; 3 Department of Electrical & Electronics Engineering, Manipal Institute of Technology, Manipal Academy of Higher Education, Manipal, Karnataka India

**Keywords:** Hotspot, Power output, Photovoltaic, Partial shading, Efficiency, Renewable energy, Electrical and electronic engineering, Energy infrastructure

## Abstract

The ever-increasing demand for sustainable energy has drawn attention towards photovoltaic efficiency and reliability. In this context, the shading and associated hotpot degradation within PV modules has become an important area of research and development. The experimental approach of this paper aims to investigate single cell shading in high efficiency monocrystalline silicon PV PERC modules. Prior to the outdoor experiment, the PV module underwent experimental testing under STC to determine variation in electrical and thermal behaviour due to partial shading. The indoor experiments are performed using Sun-simulator and the I–V and P–V curves are analysed. Further, the outdoor experiments were performed under realistic conditions. In both cases, results showed that during 40–60% shading in single cell leads to critical shading scenario causing significant drop in power output in comparison with their unshaded conditions. The maximum power loss of 36.34% and 42% is recorded for indoor and outdoor experiments. The outdoor experiments recorded hotspot temperature of 85–90.1 °C under respective 40% and 60% critical shading scenarios. The efficiency recorded in the time interval of 11:00:00 and 11:30:00 was highest for the solar radiations between 940 and 990 W/m^2^. The maximum drop in efficiency is recorded from noon till 13:30:00 time of the day. Development of hotspot is directly related to the failure or malfunction of protecting system. Hence the importance of type of PV technology, amount of shading, and critical shading scenario is presented in the study. This study is important for researcher and manufacture to consider single cell shading in PV technology.

## Introduction

**Fig. 1:**
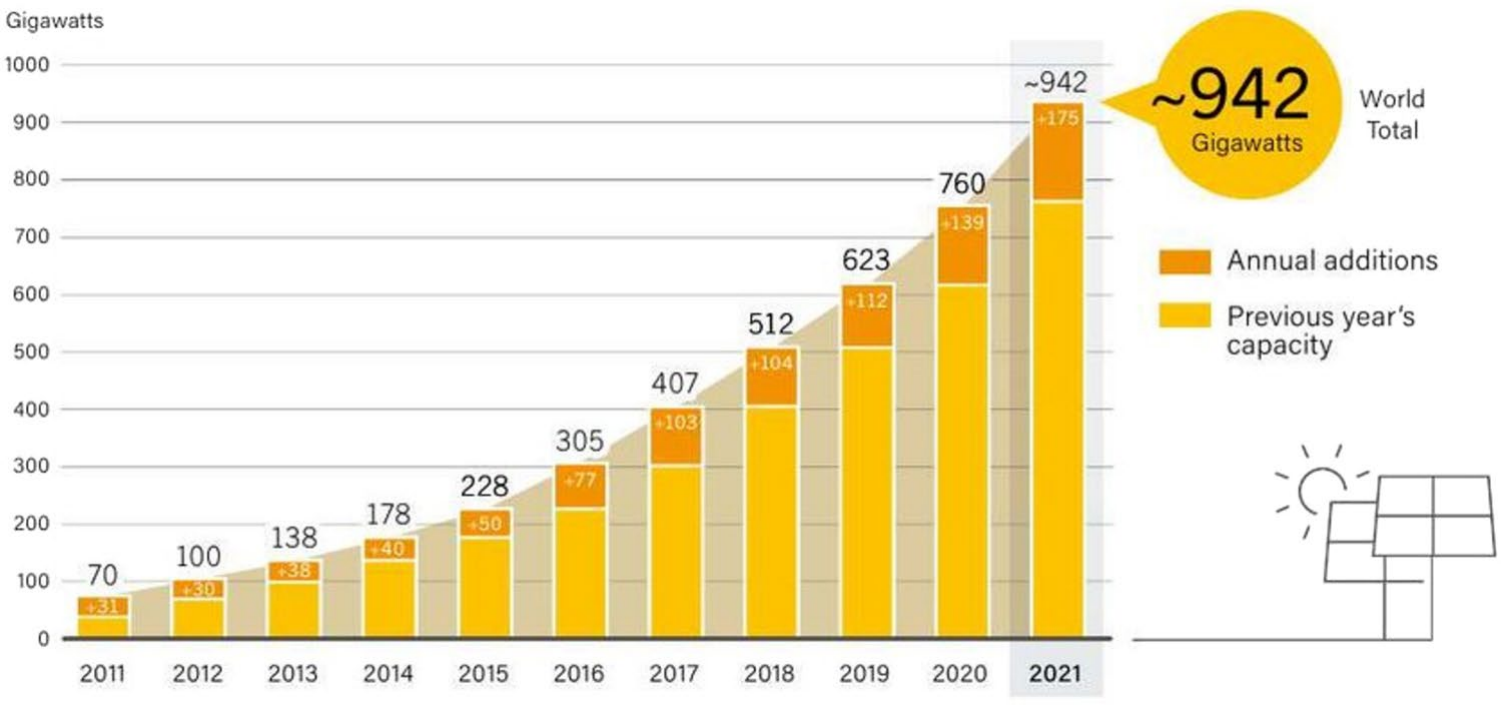
Solar PV Global Capacity and annual addition from 2011 to 2021^1,2^.

As stated in a report by “Renewables 2022, Global Status Report” the solar PV industry outshines by adding 175 Gigawatts of new capacity in 2021, as evidenced in Fig. 1. The statistical data are presented by REN 21 which is the only community at global level that provide updates, data, facts, figures, policies related to renewables. Figure 1 represent the year-by-year data giving clear picture of year wise increase in solar Photovoltaic capacity installations presented by report. Year 2021 experienced the largest ever yearly capacity increase. The total global solar PV capacity expanded at the fastest rate ever observed, summing up to 942 GW^1,2^. Three generations of PVs are available in the market including the first-generation family comprising crystalline si-solar technologies, second-generation is of thin-film technologies, and the third-generation includes emerging PVs technologies. The upcoming research in the field of various PV technologies focuses on materials and devices that are extremely efficient, environmentally friendly, economical, operate consistently, and integrate seamlessly. For PV professionals, preventing failures entirely is still the primary objective.

In this context, the degradation processes of photovoltaic systems primarily determine their lifetime and reliability. Several studies have indicated that localized overheating, or "hot spots," should receive special attention because it is identified as one of the primary causes of abrupt failures and accelerated aging. One of the primary reasons for hotspots is shading. With this aim, a methodology is developed where the behaviour of a monocrystalline solar module under shading is experimentally analysed under controlled indoor standard testing setup and outdoor climatic conditions.

**Fig. 2:**
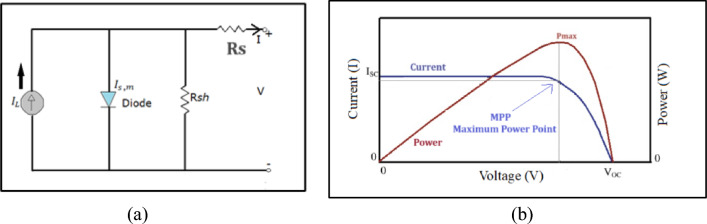
(**a**) A schematic model of a single diode solar cell (**b**) corresponding electrical characteristics of a solar cell.

A PV module is a combination of a number of solar cells together having series and parallel connections. A single-diode equivalent circuit is typically used to represent a PV cell^3,4^ as demonstrated in Fig. 2a. The light-induced current ${I}_{L}$ , the diode dark saturation current ${I}_{s}$, the diode quality factor m, series resistance ${R}_{s}$, and shunt resistance ${R}_{sh}$ are the five modeling parameters. The above-mentioned modeling parameters get affected by the variation in ambient conditions like irradiation and temperature. Changes in irradiance are reflected in the light-induced current^5^. Equation 1 represents the terminal Current (*I)* and Voltage (*V*) equation of the PV cell in terms of the above-mentioned modeling parameters.

1 $$I={I}_{L}-{I}_{s}\left(exp\left(\frac{\left(V+I{R}_{s}\right)}{m{n}_{se}{V}_{T}}\right)-1\right)-\frac{V+I{R}_{s}}{{R}_{sh}}$$

where ${n}_{se}$ is the number of series-connected cells in the PV module, ${V}_{T}$ is the thermal voltage. The I–V characteristic of a whole PV module comes from the I–V characteristics of the constituent solar cell.

A typical current–voltage (I–V) and power–voltage (P–V) curve of the cell, module, or array is shown in Fig. 2b. Figure 2b shows that both the curves I–V curve does not have any multiple steps and the P–V curve has only one peak point called the Maximum power point ($${M}_{pp}$$). These characteristics represent the standard form of the I–V curve and P–V curve. In case of degradation, these electrical characteristics tend to change^6^. Figure 2b shows three crucial points on the I–V and P–V curves: the short circuit point (*V* = 0, *I* = $${I}_{sc}$$), the open circuit point (*I* = 0,* V* = $${V}_{oc}$$), and the MPP point. An illuminated solar cell delivers a specific voltage at a given current. The operating point of a solar PV system is the combination of current and voltage values. A particular operating point of a solar cell is fixed with a load resistance. The short and open circuit conditions associated with a solar cell are defined as the load resistance equal to zero or infinitely large, respectively. The current and voltage values at short-circuit and open-circuit conditions are called short-circuit current ($${I}_{sc}$$) and open-circuit voltage ($${V}_{oc}$$), respectively. There is one specific point where the amps ($${I}_{mp})$$ times volts ($${V}_{mp})$$ generate the greatest wattage. This point on the I–V characteristic curve of an illuminated solar cell is called the maximum power point $${P}_{max}$$.

2 $${P}_{max }={V}_{mp }*{I}_{mp}$$

The additional parameter sets the maximum power generated with the product of *Voc* and *Isc* and is called the Fill Factor (FF).

3 $$\text{FF}=\frac{Pmax}{Isc*Voc}$$

The ratio of the useful output power (delivered by the conversion device) to the incident power (of the solar radiation) is called the efficiency ($\upeta$) of a solar cell.

4 $$\upeta =\frac{Vmp*Imp}{S*Ar}$$

*S* = solar irradiation (W/m^2^), ${A}_{r}$= module surface area (m^2^).

The dependability and performance of PV modules may be severely affected by the faults that develop gradually in a PV system in outdoor conditions. The electrical performance of PV modules is constrained by several factors, including the inefficiency of the cells, the discontinuity of the solar source, the unpredictable nature of the weather, and inefficient working circumstances brought on by electrical mismatch^7^. The two variables that influence I–V characteristics are temperature and solar radiation. As solar irradiance fluctuates all day long, the I–V and P–V characteristics vary accordingly. Temperature plays yet another critical factor in defining solar cell efficiency. This paper aims to understand how the attributes of the I–V and P–V curves get affected due to shading, specifically in Mono PERC PV modules under STC conditions and outdoor conditions. Thereafter, to conclude the potential development of hotspots in PERC monocrystalline PV technologies. A research study presented by authors DC Jordan et al., presented that hotspot are major concerns over past decade in installed PV system. Single cell shading is very crucial point to consider as it is related with turning ON of protecting during hotspot development in a PV system. However, it is been observed that a vast study is been done either on small wattage solar panel and individual solar cell inside laboratory or the research is been conducted on a commercial large scale PV system. As mentioned in the report^8^ with almost 60% market share, PERC design is consolidating its supremacy in commercial field. Hence it calls attention to research the impact of dynamic environment on thermal and electrical behaviour of commercial designs high efficiency PERC PV modules due to single cell shading. The study targets to finds out the critical shading area for a single cell in case of commercial PERC modules that could cause hotspot to develop. The extended part of the research finds out the maximum efficiency and duration when the maximum efficiency is achieved in several shading percentages. Hence the work provides following key contributions as:

1. Critically analyses of the environmental impacts on high efficiency commercial monocrystalline PERC PV Module and analyse the vulnerability of PERC technology towards partial shading and hotspot development.
2. Electrical and thermal responses PV Modules as well as the severity of shading of single cell in high efficiency PERC Modules.
3. With the aid of data acquisition systems, the experiments are continuously monitored and the various readings of temperature, output power, efficiency is recorded during the day.
4. The influence of type of PV technology, amount of shading, critical shading percentage is presented in the study. This study is important for researcher and manufacture to consider single cell shading in PERC modules. Based on the recorded results a novel technique could be researched in future studies to improve the protecting circuit against hotspot formation.

## Literature review

### Shading and its impact on PV module

Understanding the efficiency, reliability, and durability of photovoltaics is very important in perspective with the rapid growth of PV systems. A PV system’s performance is directly affected by shading. Shading can be in any form—complete shadow or partial shadow. The shaded portion of the illuminated PV module acts as load resistance and starts to consume the electrical power. In such conditions, the unshaded parts of the PV module compel the shaded part to go in reverse bias condition. Under reverse bias conditions, the shaded portion dissipates power, causing surface temperature to increase and consequently leading to hotspot. A bypass diode is therefore needed to prevent irreversible damage to the shaded solar module. The most frequent causes of shadowing a PV module are vegetation, buildings, the build-up of dust and soil, and animal and bird waste. A shading defect caused by any of these reasons leads to a drop in generated output power. Entire PV panels in the array will be impacted if a single cell or single PV panel experiences shading. Therefore, it's crucial to work on how to lessen the impact of shading on PV systems.

**Fig. 3:**
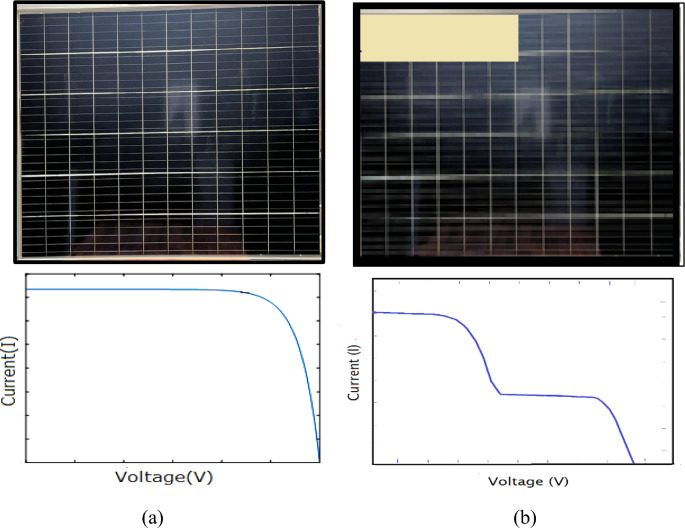
I–V characteristics of a module (**a**) Uniformly illuminated (**b**) Partially shaded.

There has been intensive research done to understand the on how the shading is impacting energy of a PV system^9,10^. Figure 3 gives a brief demonstration of I–V curves in two different scenarios: uniformly illuminated PV module and partially illuminated PV module. The degradation caused by shading is meticulously presented in a research paper by M. C. García et al. The work done in their research demonstrated the diode characteristics under shaded conditions, where a PV cell tends to function in the 3rd quadrant, indicating the power output response of a shaded module^11^. Another research work demonstrated that the partial shade hinders the PV performance and damages or degrades the module over time^12^. According to Lappalainen et al., the partial shading is primary reasons of mismatch losses observed in a PV system. The sharp shadows create the largest mismatch loss. These types of shadows are major issues on the roof top as well as residential installations^13^.

Partial shadows created by moving clouds are of great concern in large-scale PV setups, creating mild irradiance variations among adjacent PV modules. Mohammedi A. et al., 2014 presented the complexity and substantial impact of shadowing. Their research work showed that several partially shaded arrays can impact the output more severely than one completely shaded array^14^. Another research article confirmed the power loss of up to 10% to 70% due to shading^15^.

The connection configuration in a module is one of the significant factors that impact the outputs under shading. However, Vigni et al., 2015 demonstrated that incorporating an extra DC/DC converter and modifications in the configuration of the PV system could upscale the array's energy yield by 30% and power losses by shading can be recovered by 13%^16^. The authors presented different configurations of series–parallel combinations to lessen the effect of partial shading. However, the effect of by-pass diode was not considered^17^. The research article by Indu et al. proposed a novel Su Do Ku arrangement, which could effectively reduce the effect of shading in a PV system^18^. The bypass diode can prevent a cell voltage from reaching its breakdown voltage while shading. However, Rossi et al. in their experiment concluded that detrimental effects due to shading, such as hotspots, still may appear even if the by-diodes are incorporated into the module^19^.

A research article proposed a novel topology incorporating BJT with two MOSFETs instead of using a bypass diode to maintain the voltage positive across the shaded cell. This advancement could successfully lower the power loss due to shading. However, the model complexity and overall system cost increased^20^. The shading can cause the variation in the temperature at cell and module level. The result of a research study demonstrated temperature surges and decrease in output voltage under various shadow situations^21^. Software-implemented research is also one of the approaches used to analyze PV modules under shading. Several methodologies have been identified in recent research such as artificial neural networking to recognize the short circuit types of faults in a PV array^22^ while few other authors incorporated fuzzy logic for the same purpose^23^. Software-employed simulation-based models, such as PSpice, PSIM, EMT, and others, are used thoroughly to investigate shadowing effects on photovoltaic systems^24,25^. Though quite a large literature is available for partial shading in conventional field installed modules, addressing partial shading in BIPV is still a new emerging research area. Authors Q. Li et al. presented their remarkable work where a multi-physics mathematical model is presented to foresee the performance of electrical, thermal and structural parameters under three conditions like no-shading, shading and masking and predicts when the hot-spot occurs in BIPV technology^26^. Yet a significant research regarding power output prediction in partially shaded BIPV is presented by L. Zhu et al. where the author proposed a thoughtful approach that correlated the solar cell shaded area proportion (%) with photocurrent and series resistor. Based on this analysis, a simplified mathematical model is presented to predict the various parameter under the partial shading conditions. Secondly, they demonstrated relationship between shaded area proportion (%) with I–V and P–V results of PV Module and finally descried the method to maximise the power output of BIPV Module^27^. The present literature addresses the issue to partial shading and give us an idea how research in this field can lead to improvement in performances of different PV technologies.

### Hotspot in photovoltaic

Generating hotspots in PV systems is one of the most challenging conditions. Hotspots can be understood as localized heat sources that may develop in any part of a PV module, leading to an increase in temperature in a particular area. Many reasons are pointed out in the available literature for the development of the hotspot, such as sudden changes in the day-to-night temperature transition. One of the significant reasons for hotspot development documented in the literature is “shading”^28^. The appearance of I–V curve provides much information about hotspot heating^29^. Due to their frequency and severity, hotspot has emerged as one of the prevalent modes of degradation in installed PV systems^30^. However, some authors used a bypass diode to reduce the hotspot mechanism. The concept of bypass diode was initially proposed in the year 1982 to reduce power loss and hotspot conditions^31^.

The temperature in hotspot areas may even rise over 100 °C, which further leads to other degradations like breakage of glass and browning discoloration, and it may even induce fire in severe cases^32^. A research article reported that partial shading is more dangerous than full-cell shading. The paper proposed a new approach to identify the shading in a PV module and, hence, knowing the stage where the cell is destroyed permanently due to localized heating^19^. In their article, the author showed the numerous ways to recognize the impact of shading and hotspots. They also demonstrated the impact of non-uniformity of solar radiation using infrared techniques^33^. The research study showed that shading ratios from 40 to 60% potentially increased the temperature of the shaded portion from 25 to 105 °C, respectively^34^. Even the new design technology is facing hotspots due to shading responses. In an experiment, shingled modules showed a greater tendency to build a hotspot than half-cut modules when both modules are exposed to the same shading responses. The hotspot of 145 °C temperature was observed in the study, along with the variation in the cell characteristics during shading^35^. In addition, the presence of shading creates the chances of generations of hotspots that may cause the development of multiple peaks in the cell characteristics that directly impact the output generations and drop the life expectancy of the PV module^36^. With the technology advancement there are many cutting-edge techniques aim to increase the resilience of PV technology in various conditions including shading. In one of the recent research studies, the authors proposed a novel circuit design to mitigate the hotspot in PV Module by employing a dual stage current comparator. Through this technique, the hotspot temperature of 55 °C dropped to 35 °C and the output power increased by 5.3%^37^. Another novel circuit for hotspot mitigation is proposed by authors Ghosh et al. where the circuit named as HSMC- Hotspot Mitigation Circuit is capable of reducing elevated cell temperature by 10 °C under mismatch conditions by modified bypass techniques^38^. Such a technique could be very beneficial for large scale PV systems potentially reducing mitigation process. Recent literature presented an article on optimization technique called as a black widow reconfiguration which proposed to boost power output and better functioning scenario in partial shading conditions. The technique showed better power generation in dynamic and static partial conditions and mismatch losses get minimised with its implementation^39^. All these progressions together aim addressing the problem of hotspot and mitigating the development of hotspot.

#### Fundamental details to estimate hotspot temperature

Reverse biasing of a solar cell leads to power dissipation that could accelerate ageing and increase failure rates in PV module^40^. The hotspot temperature can be estimated as described below^41^ through result could be technology specific as well^42^:

For m number of PV cells in a string protected by a diode of a PV module operating under S irradiance with ${T}_{cell}$ be the cell temperature, Voltage be *V* and Current be ${I}_{C}$, a condition of hotspot develops in a cell when its ${I}_{SCD}$ that is short circuit current decreases due any internal and external factors as shown in Eq. 5:

5 $${I}_{SCD}<{I}_{C}$$

Consequently, the hotspot-affected PV cell inclines to work at a negative voltage:

6 $${V}_{df}= -\left(n-1\right) {V}_{ndf}+V$$

where *df* and *ndf* in the subscript representation defective (hotspot affected) and non-defected PV cell respectively. $\Delta {T}_{hs}$ is the increase in temperature in the defective (hotspot affected) as compared with healthy cell (non-defective cells). The $\Delta {T}_{hs}$ is directly related to the product of ${I}_{C}\times {V}_{df}$.

$\Delta {T}_{hs}$ can be calculated as:

7 $$\Delta T_{{{\text{hs}}}} \approx {\text{C}}_{{\text{T}}} \times \frac{{IC \times V_{df} }}{A}$$

where *A* is the surface area of the cell, ${C}_{T}$ denotes the thermal dissipation coefficient. ${C}_{T}$ can be calculated using Eq. 4. The NOCT is the nominal operating cell temperature.

8 $$C_{T} = \frac{NOCT - 20}{{800 \;{\text{W/m}}^{2} }}$$

#### Electro-thermal modelling of a PV cell under hotspot condition

**Fig. 4:**
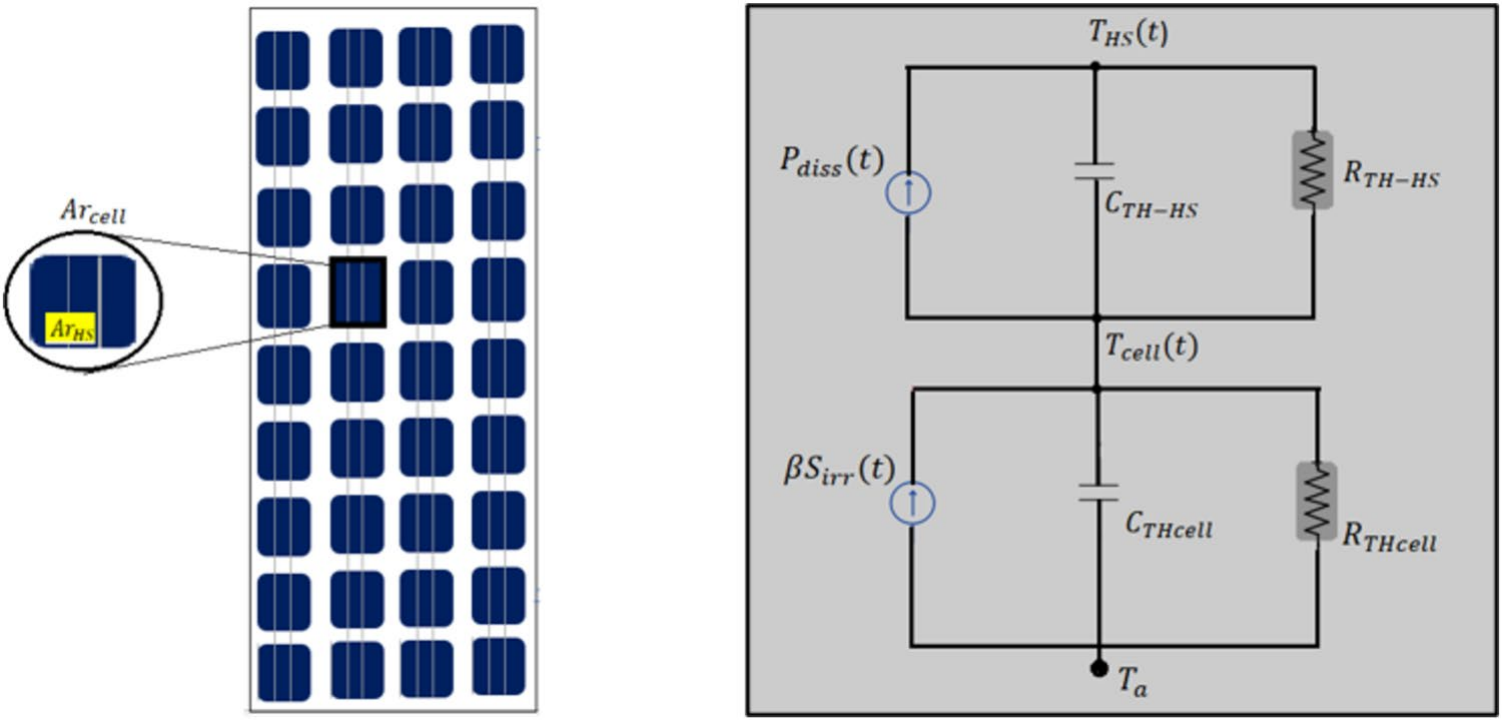
The equivalent thermal model to estimate the temperature of the portion of PV cell ${(Ar}_{HS}$) under hotspot conditions with time.

Thermal modeling provides clarity in finding the temperature of the shaded area ( $${T}_{HS}$$) of the module under hotspot $${(Ar}_{HS}$$) while the entire area of the PV cell is denoted by $${Ar}_{cell}$$. The time “*t*” is the instant at which the PV module undergoes the shading condition, and the $${t}_{HS}$$ is the generic time instant (reverse bias state), where the PV cell undergoes hotspot conditions. The thermal model can be used to determine the thermal behaviour of a shaded PV cell. Figure 4 represents the equivalent thermal model to estimate the time dependence of the temperature of the portion of PV cell $${(Ar}_{HS}$$) under hotspot conditions. Two series of interconnected RC circuits are present in the thermal model. The upper RC circuit in Fig. 4 demonstrates the temporal functioning $${Ar}_{HS}$$ during full or partial shading as a function of $${P}_{diss},$$ the power dissipated in the shunt resistor $${R}_{sh}$$, shunt resistor. The lower RC circuit explanation the temporal functioning of the PV cell temperature as a function of Solar irradiation ($${T}_{cell})$$ only.

The expression for computing ${T}_{HS }(t)$ is explained in the equation:

9 $${T}_{HS }\left(t\right)={T}_{a}+{R}_{THcell} {s}_{irr} \left\{t< {t}_{HS}\right.\}$$

10 $$\begin{aligned} T_{HS } \left( t \right) & = T_{a} + Ar_{cell} R_{THcell} S_{irr} \left( t \right)\left( {\beta + \left( {1 - \beta } \right)e^{{ - \left( {\frac{{t - t_{HS} }}{{R_{THcell} C_{THcell} }}} \right)}} } \right) \\ & \quad + P_{diss} R_{TH - HS} \left( {1 - e^{{ - \left( {\frac{{t - t_{HS} }}{{R_{TH - HS} C_{TH - HS} }}} \right)}} } \right)\quad \{ t \ge t_{HS} \} \\ \end{aligned}$$

where β = $$\frac{{S}_{irr-shaded}}{Sirr}$$ shows the relative mismatch condition in the solar irradiance between shaded ($${S}_{irr-shaded})$$ and the uniformly illuminated cell that is unshaded ($${S}_{irr}$$).

Equation (9) represents the condition before a hot spot condition develops ($t< {t}_{HS}$); during this moment, the temperature of the whole module PV cell is constant and can be seen as ${T}_{HS}$ = ${T}_{cell}$ ${T}_{a}+{R}_{THcell} {S}_{irr}$. In this condition, the PV cell works under Solar irradiation ${S}_{irr}$ and represents solar PV cell in forward biased condition. Hence, power dissipation is negligible, and cell temperature depends only on Solar irradiation,${S}_{irr}$. ${T}_{a}$ is known to be the ambient temperature of the module.

Equation (10) illustrates the shaded PV cell entering hotspot condition (*t* ≥ ${t}_{HS}$). Under this condition, two phenomena are noticed: the shaded PV cell is impacted by reduced solar irradiation,${S}_{irr},$ and contribution of power dissipation through ${R}_{sh}$. The reduction in solar irradiation tends to reduce the temperature with time, while the power dissipation causes an increase in temperature. It has been well observed in the available literature that the power dissipation through ${R}_{sh}$ is more dominant than primary phenomena causing temperature ${T}_{HS }$, to rise very quickly.

The parameters in the above equation ${T}_{HS , }{R}_{TH-HS}, {C}_{TH-HS},$ represents temperature, thermal resistance and thermal capacitance given in [°C], [°C · m^2^/W], [°C · m^2^ · s/W] respectively, of the PV cell of area ${Ar}_{HS}$ under going hotspot. ${T}_{cell }[^\circ \text{C}],{R}_{THcell}$, [°C · m^2^/W] $, {C}_{THcell}$ [°C · m^2^· s/W] represents temperature, thermal resistance and thermal capacitance respectively for the rest of the PV module area not under hotspot. The parameters ${R}_{TH-HS}, {C}_{TH-HS}$ depend upon the materials incorporating the top layers of the module cells.

The literature makes it evident that a significant study has been performed on impacts of partial shading in a PV Module.

## Methodology

Combining laboratory and outdoor testing is helpful to ensure that PV modules meet their performance requirements and consistently produce power over their operational lifetime. Different PV technologies tested inside a laboratory may behave when installed in outdoor conditions. This study aims to analyse how a commercial PV module behaves when placed in outdoor conditions. Partial or full shading of PV systems is a widespread phenomenon. However, shading could generate non-linearities in electrical characteristics of PV systems. The initial investigation of the PV module is done in the laboratory under STC conditions. Under standard conditions, different shading percentages are applied to a single PV cell, and the responses of the PV module are recorded. The experiment records the obtained variations in: electrical characteristics, performance parameters, temperature of the shaded cell, and temperature of the entire PV module due to shading.

The results of shaded and unshaded are recorded and analysed. A module may behave very differently when installed in outdoor conditions. Due to variable solar radiation and ambient temperature, the shading may impact the efficiency and performance of photovoltaic modules Under fielded conditions. Hence, during outdoor testing, the impact of shading is analysed under various shading scenarios. After that, possibilities for the development of hotspots are analysed. Along with this, various electrical parameters and electrical characteristics are noted. It should be noted that for some temperature directly depends on concentration of leakage current. The temperature increase is influenced by the region where the power is dissipated^11^. The outdoor experiment will present a thorough impact on thermal and electrical properties due to shaded PV cell. The operation of diode conduction depends on two phenomena—the amount of shading and the shape of the I–V characteristic obtained under a shaded scenario. The measured temperature of the shaded cell through the day is important to attain a corelation between shade-affected PV cells, bypass diode, and increase in temperature. Furthermore, experiments performed in the laboratory will provide basic information about the possibility of hotspot occurrence in shaded cells subsequently supported by outdoor testing. The detailed experimental setup is explained below.

The experimental approach adopted to determine the corelation between outdoor behaviour and flasher values under standard test settings. This involves repeated laboratory and field measurements under different partial shading conditions. This method seeks to examine the connection between the STC warranties and real-world experiences for commercial PERC monocrystalline technology. Terawatt Testing and Research Institute Private Limited, Rajasthan, is renowned organization that provides the accredited solar testing indoor and outdoor laboratory where all the measurement procedure are performed with complete quality management system as per ISO/IEC-17025 standards.

The I–V and P–V measurements are recorded during indoor experiments with a steady state G-Sola AAA sun simulator capable of generating variable irradiations from 400 to 1100 $\text{W}/{\text{m}}^{2}$. The simulator is designed as per IEC 60904 guidelines and meets international guidelines. The entire system has 3 units, the module test container (MTC) where the module is placed, pulse light generator (PLG) which generator the pulse for flashes and I–V test operating system. The I–V test system has an operating software, LabVIEW, to record and present various I–V and P–V curves. An electronic load and professional data acquisition system is also present to trace the graph and record all the experimental results. The simulator makes use of xenon lamps as a source of light generator to stimulate natural sun light for testing purpose to match AM 1.5 spectrum. In the present study, experiment is conducted with fixed irradiation of 1000 $\text{W}/{\text{m}}^{2}$ and variable shading percentages on a single cell. The I–V and P–V curves, ${I}_{sc}$*, *${V}_{oc}$*,*${P}_{max}$* , ɳ* are generated and recorded through the software system as a procedure of the experiments. During the test, the PV module is exposed to flash test analysis, which will confirm the accuracy as mentioned in the datasheet. After that, the PV module is tested with the flash test for different partial shading conditions which is explained next section.

The outdoor work intends to form a bridge between STC data collected from the laboratory and yield of the partially shaded PV module. Though experiments performed indoor offers a highly controlled setup, the results may differ in the external environments due to varying ambient conditions. To understand the partial shading and hotspot development further, an outdoor experimental study is performed. The module is tested to analyse the electrical and thermal response due to single cell shading. All the variables such as solar irradiation, daily ambient temperature, wind and humidity are recorded continuously and saved with the automatic data loggers available at the outdoor setup. At present this is the best methodology adopted to understand the development of hotspot due cracks, mismatch caused by partial shadings.

### Indoor experimental setup

**Fig. 5:**
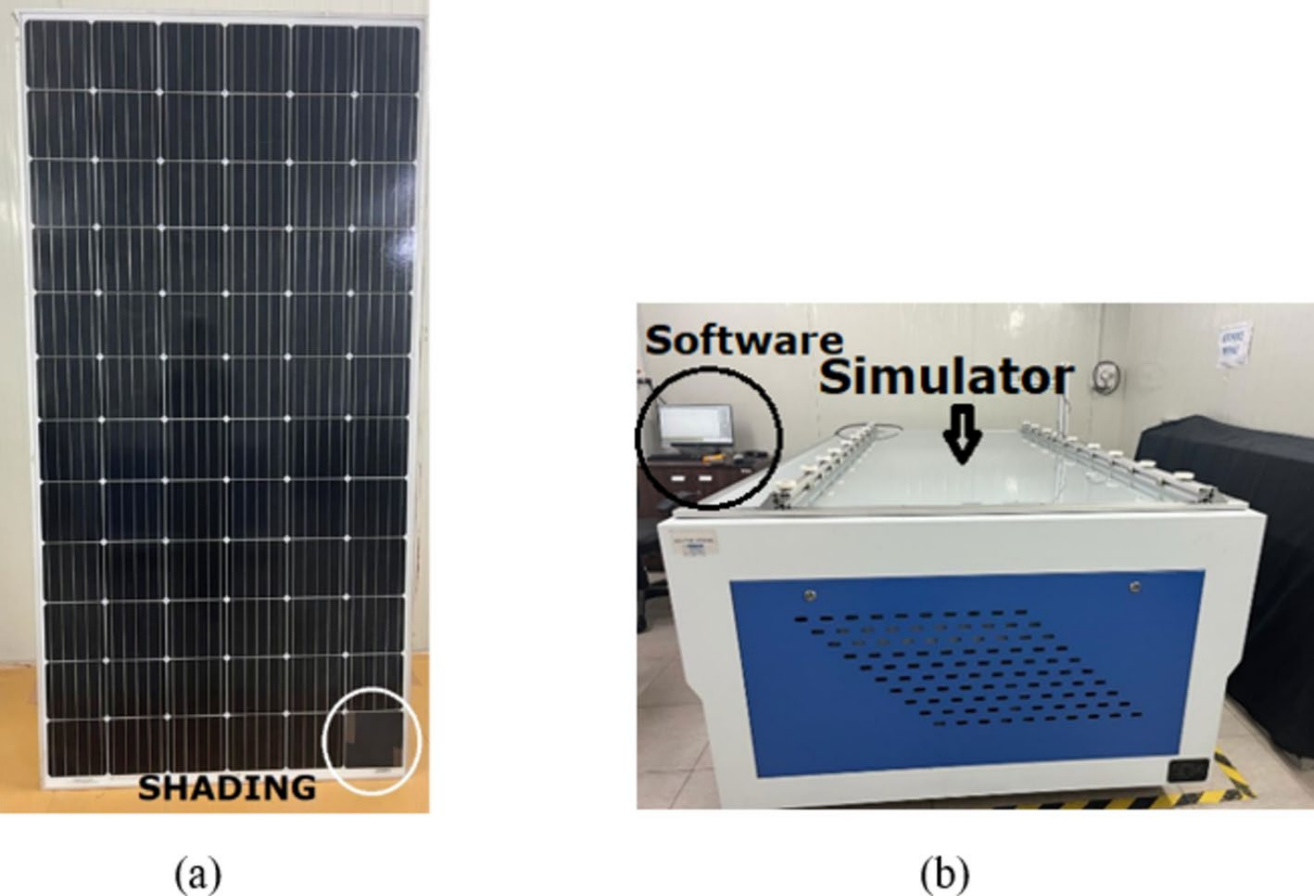
(**a**) reflect a 72-cell monocrystalline PERC PV module representing the method and material used for shading. (**b**) shows the PV module positioned on the Sun Simulator for experimental purposes.

The location for the experimental setup is the Terawatt Testing And Research Institute (TTRI), Khushkhera, Bhiwadi, Rajasthan, India. The indoor laboratory setup is shown in the Fig. 5. The laboratory has a well-designed experimental setup with a Solar simulator Class-AAA, providing variable irradiations ranging from 400 till 1100 W/m^2^, Data logger with LabVIEW software that extracts I–V and P–V curves, Temperature gun -Fluke model. The electrical specifications of the PV module are given in Table 1. Various shading profiles are created using an opaque black rubber piece, as shown in Fig. 5. The opaque black rubber piece was positioned on the immediate surface of the cell. The sun simulator is used to create an artificial environment of the sun and, along with it, a software incorporated with it. This setup provides information on electrical characteristics and parameters like output power, efficiency, and fill factor. Figure 5 gives the indoor setup at the PV testing laboratory.

**Table 1:** Module specification.

PREL -370W (monocrystalline PV module)	
Rated peak power (W)	370 Wp
Open circuit voltage	48 V
Short circuit current	9.90 A
Fill factor	77.82%
Efficiency	19.57%
Junction box	IP67/IP68 3 bypass diode
Cell encapsulant	EVA (ethylene vinyl acetate)

#### Experimental procedure at Standard conditions

For PERC, monocrystalline PV panel experiments are performed in two steps. The STC conditions are characterized by 1000 W/m^2^ of solar irradiance with cell temperature of 25 °C.

I. Primarily, the experiment is conducted under no shading conditions. The electrical parameters and characteristics are recorded. This is done to verify that the module is in healthy condition and does not pursue an initial defect.
II. Secondarily, the shading is applied from the bottom to the top of the module. The shading starts by covering 20% of the cell area, then 40%, 60%, and 80%. Finally, the complete shading is done by covering 100% of the area of the selected cell.

**Fig. 6:**
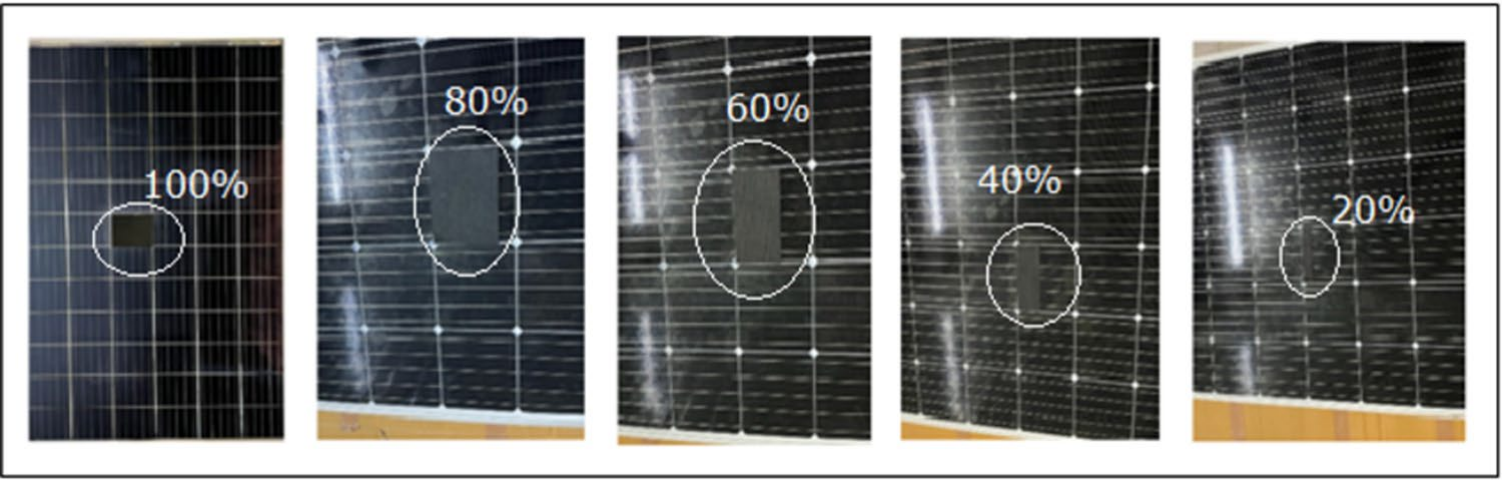
Shading percentage on single PV cell by using black rubber piece.

**Fig. 7:**
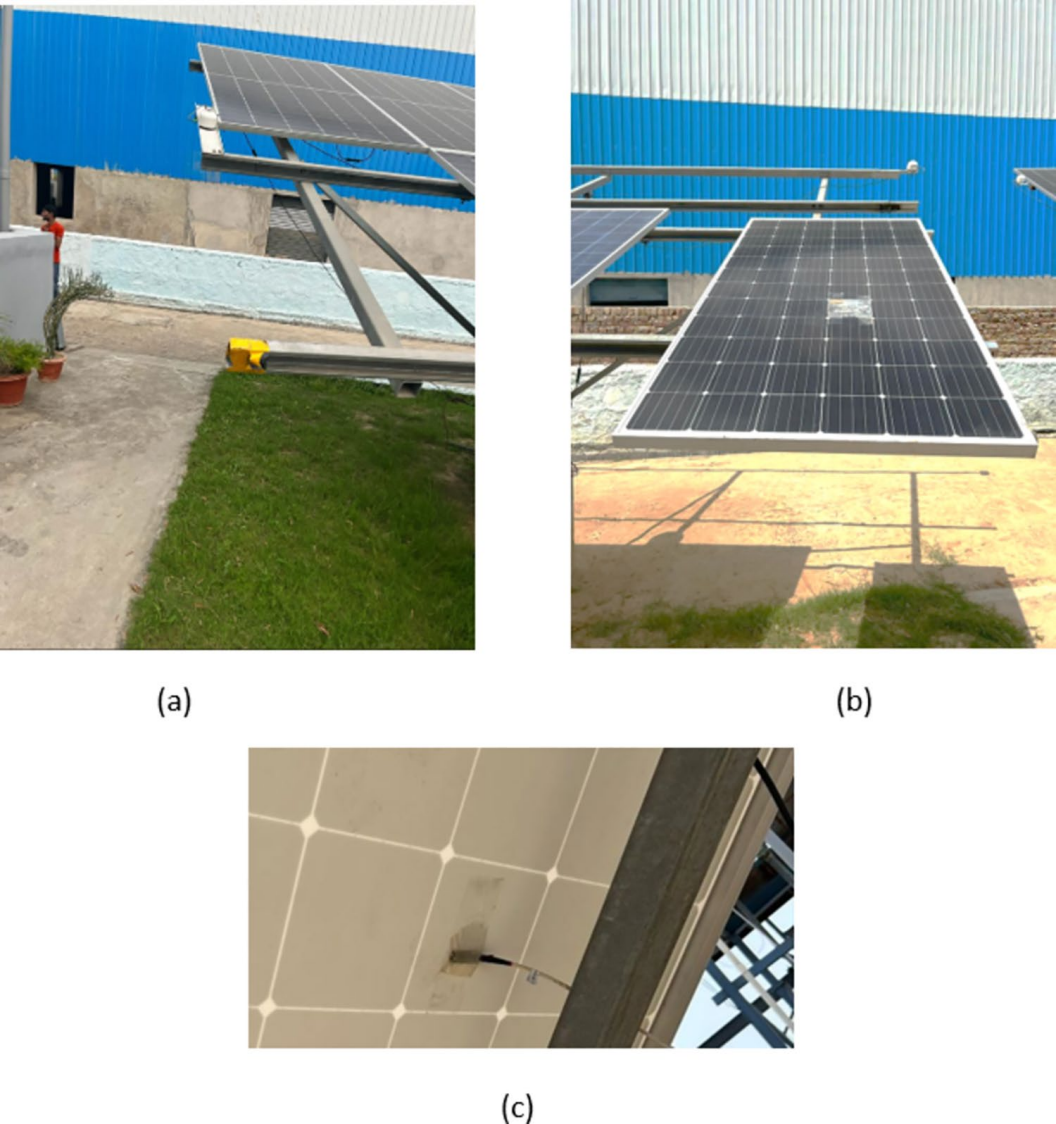
The figure depict the arrangement of outdoor experimental setup, (**a**) represents the placement of pyranometer and thermocouples attached at the back side of the module to record temperature elevation (**b**) show the alignment of the module during experiments (**c**) Attachment of thermocouple.

**Fig. 8:**
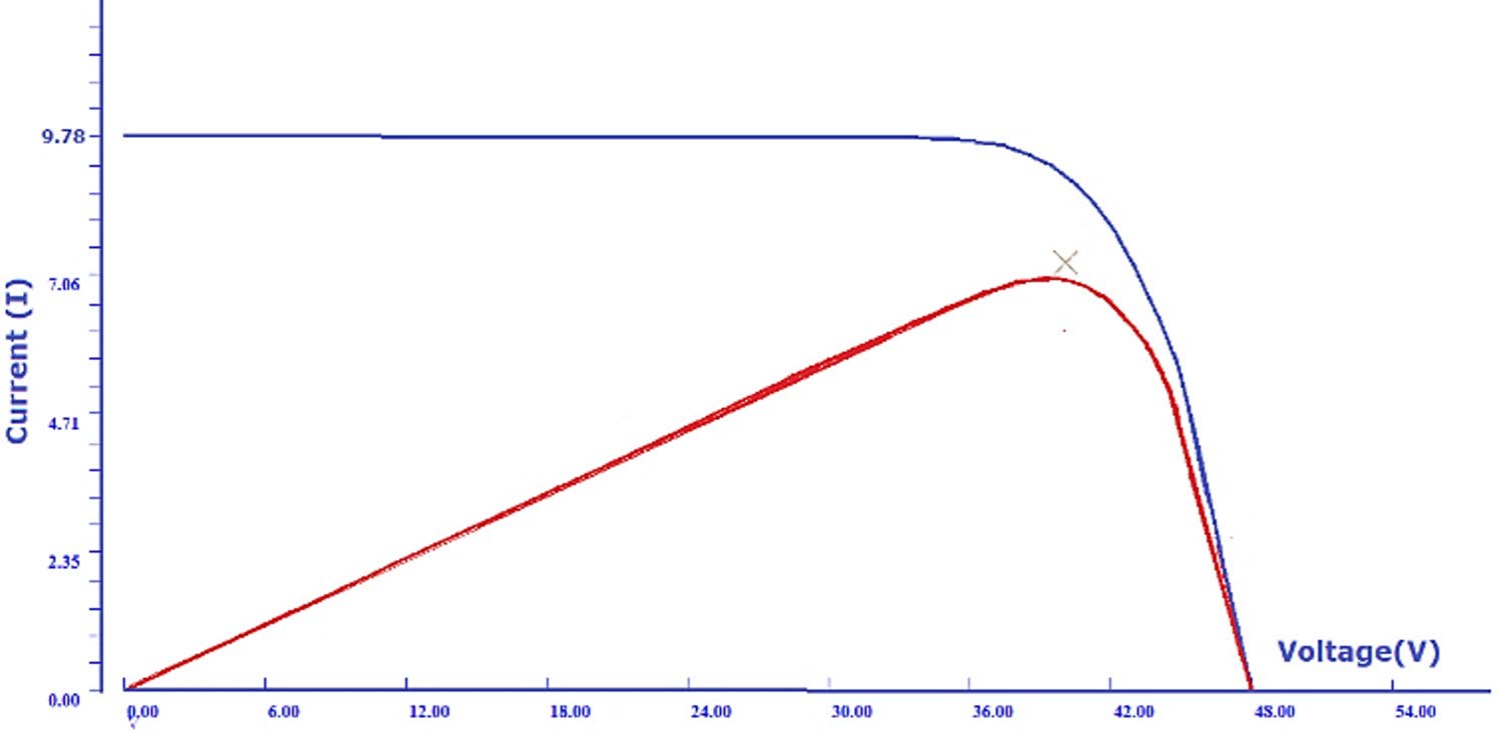
The generated I–V and P–V characteristics of PV module at standard testing conditions.

**Fig. 9:**
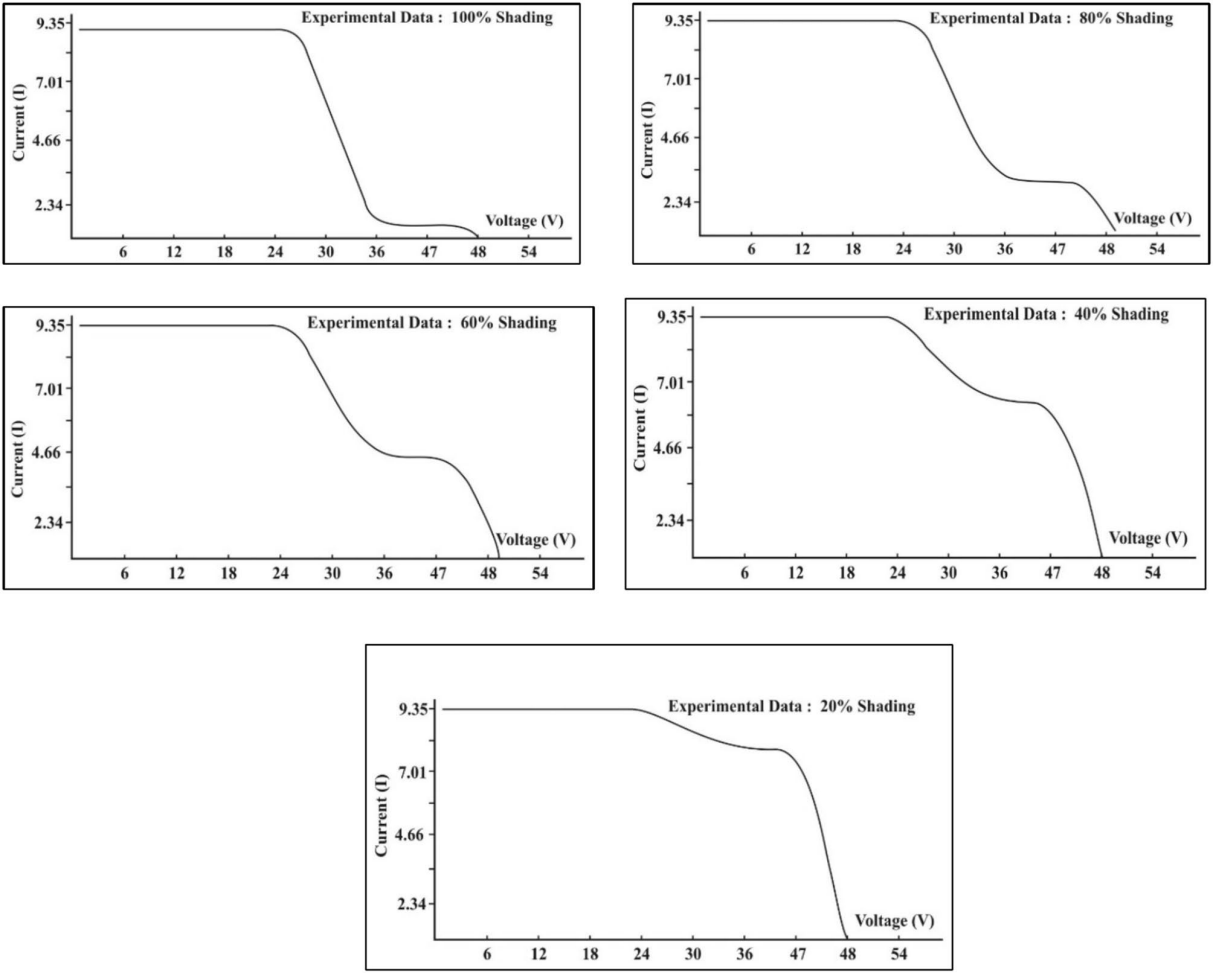
Module I–V response under different shading percentages.

**Fig. 10:**
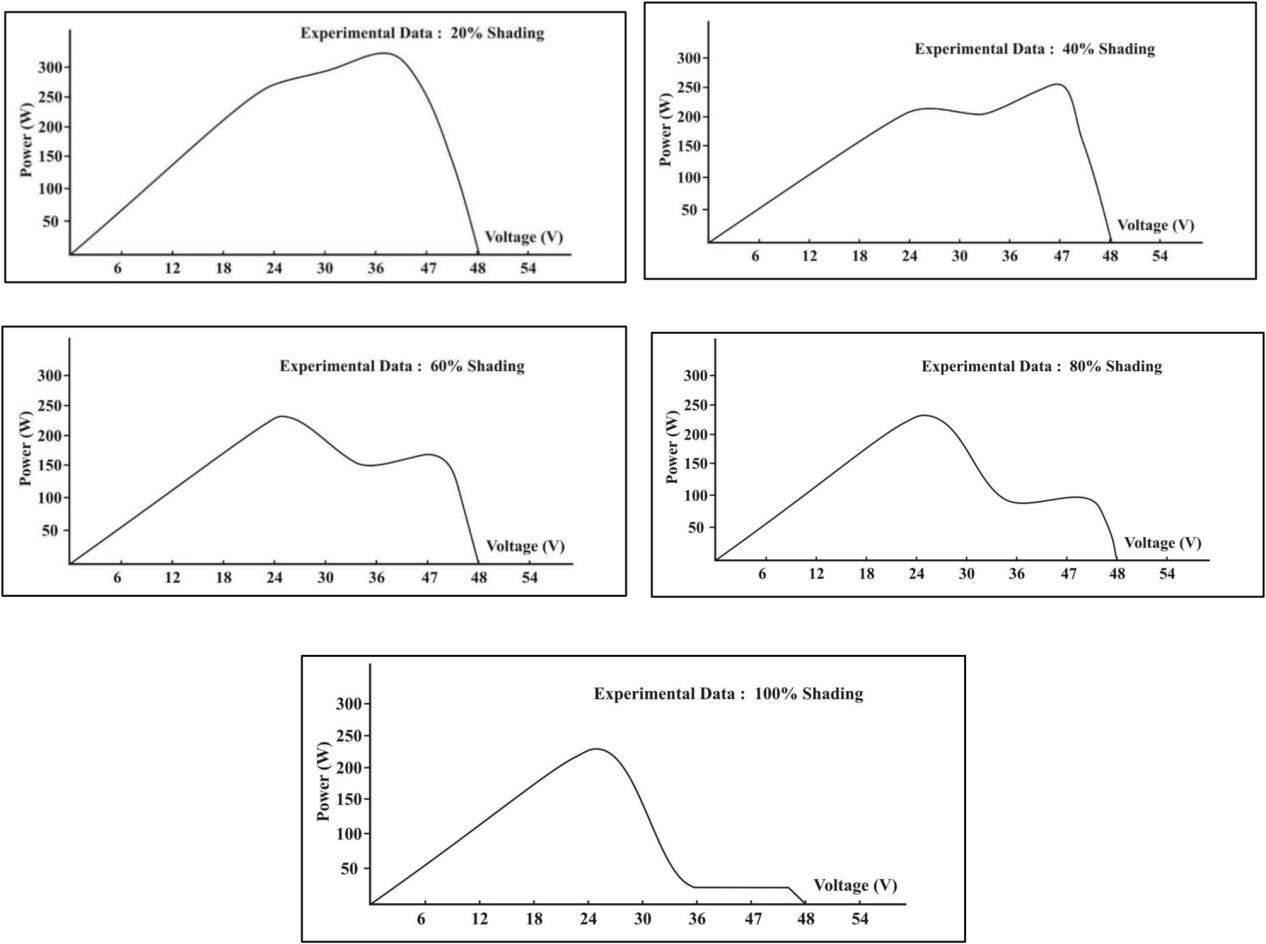
Module P–V response under shading percentage.

The implemented shading pattern is shown in Fig. 6 and the arrangement of outdoor experimental setup is explained in Fig. 7. Their respective electrical characterization results are demonstrated in Figs. 8, 9 and 10, respectively.

### Outdoor experimental setup

The experiment is conducted in the outdoor testing setup at Terawatt Testing and Research Institute (TTRI), Khushkhera, Bhiwadi, Rajasthan, INDIA. The installation setup has a bracket when module is placed at south facing angle at a tilt of 28°. Two K-type thermocouples are attached at the back side of the module to get the reading of shaded and unshaded cell during the experiment. On the top of the bracket, a pyranometer is installed at 28° of inclination to get the readings of varying solar irradiance. The entire setup of the PV system is attached to an automatic data logger that tracks and record the measured data. The various environmental conditions like humidity, ambient temperature, and wind speed are monitored with the sensors installed at the institute; however, such readings are not required for this experiment. Usually, these measurements play a significant role in PV predictive modeling, hence eliminated in the presented papers.

All outdoor experiments are conducted on the mono PERC module, which is initially tested in indoor conditions in the lab. The outdoor experimental studies are conducted in the month of April, 2023. The weather condition during this month in Bhiwadi, Rajasthan is typically dry and hot climate. The average relative humidity during the days when experiments were conducted is noted to be approximately 20.6%. The precipitation was minimal round the month of about 0.02 mm which show very dry conditions. The outdoor experiments are performed on a clear and sunny day with an average ambient temperature for the month noted to be + 34 °C and the average wind speed for the month is 1.5 m/sec. The readings are noted for every single minute for the whole day, starting from 08:15:00 to 17:30:00; however, the results are analyzed from time 9:00:00 to 16:00:00. The average solar irradiation noted at 9:00:00 is 560 W/m^2^ and 470 W/m^2^ at 16:00:00 during the days when test is performed. However, these values keep on increasing as the day passes by, reach a peak value, and drop by post afternoon. It is also observed that there is not much variation observed in the irradiance during the noon time as the angle of inclination of sun rays is smallest around 12 noon.

## Results and discussion

First, the experiments are conducted in an indoor laboratory setup under STC conditions. The different shading profiles are implemented by covering 20%, 40%, 60%, 80%, and 100% of the cell area. After that, the experimental analysis is performed outdoors with the exact shading percentages starting from 20 to 100% and applied on five consecutive days respectively. The results are noted down and discussed in the subsections below. All the measurements are recorded according to standards and under ISO/IEC-17025 accredited laboratory^43,44^.

### Experimental analysis of indoor testing

#### Electrical characteristics at standard test conditions with no shading

Primarily, the test is conducted on a fully illuminated, unshaded module PV module at STC (indoor)conditions. Figure 8 shows recorded I–V and P–V curves of the module at standard test conditions. The blue graph shows the current–voltage curve, and the power–voltage curve are shown by red curve. For the I–V curve, the x-axis shows the voltage (V) whereas the y-axis shows the current (I) measurements. Likewise for P–V curves, the x-axis shows the voltage (V) and y-axis shows the power (P) measurements. Figure 8 demonstrate the curves to be in perfect shape and no deviations from standard shapes. The recorded values of short circuit current is 9.81 A, open circuit voltage is 48 V, and maximum output power is 364.822 W. The other performance parameters, which are efficiency and fill factor, are available in Table 2.

**Table 2:** Performance parameters of the module tested at STC.

Condition	Shading	Pmax (Watt)	Efficiency (η)	Fill factor (F.F.)
STC	No shading	364.822	18.55%	76.14%

#### Electrical characteristics at standard test conditions with shading

PV modules with shading defects can be easily traced with I–V and P–V curve characterization. Numerous studies have demonstrated the occurrence of multiple steps in I–V characteristics and multiple notches in P–V curves of the shaded PV modules^45,46^. Multiple local maxima were found in simulation and experimental studies conducted on a 72 mono-crystalline cells 180 W PV. The author found that such a condition is very dangerous and can source permanent damage^47^. Even type of distribution of shadow can cause damage to the PV Module. In this context Bayrak et al. 2017 demonstrated the influence of vertical, horizontal, and cellular shading on the output of PV modules^48^. The corelation between shaded area and its influence on output is presented by Galeano et al. 2018. Their findings showed that rise in shadow coverage influences the standard shapes of the I– curve and minimises the maximum output current^49^. Similar results were obtained in a study where the results showed the occurrence of multiple peaks in the I–V and P–V curves with cellular and vertical type shading but no variation in the curve with horizontal shading^50^. Authors Lyden and Haque presented a methodology to estimate the influence of partial shading for operational PV systems. The results confirmed the visible multiple global maximum power points due to shading under rapidly changing irradiance conditions^51^. The authors in their work analysed the impact of shading generated by buildings shading, wire poles shading, plants and birds droppings shading on 20 MWp grid-connected photovoltaic project in northwest China and found similar distractions in electrical characteristics^52^. A study analysed the PV system under shaded with using MATLAB/Simpower system software. The author demonstrated the effect of shape, intensity and distance of shadow on a PV system^53^. Figures 9 and 10 exemplify the recorded results under different shading profiles, respectively.

#### Interpretation of electrical characteristics obtained from indoor experiment

The I–V and P–V curves represents the electrical characteristics of PV module. These curves have a standard shape under healthy working conditions. Any kind of faults in PV module, distorts I–V and P–V curves from their actual shapes. The graphs in Figs. 9 and 10 show visible steps and notches in I–V and P–V curves, respectively. According to literature, the results in Figs. 9 and 10 are clear indications of mismatch due to shading in the PV module^47,54–57^. The maximum power point (MPP) in a PV system, method monitors the instances where the maximum power is produced. Under no-fault conditions, there is just a single maximum point, as demonstrated in Fig. 8. While under shading, the PV panel has multiple operating points, which can be seen in this paper as well in Fig. 10. Under such conditions, the MMP tracker finds it challenging to distinguish a single GMMP (Global Maximum Power Point) from several local maxima (peaks). Its nature of unique global peak is defined by array configuration and shading pattern.

Due to shading in a single PV cell, the short circuit current ${I}_{sc}$ shows minor drop in its original unshaded values. Hence, the ${I}_{sc}$ of the PV module is affected due to shading. A similar effect on short circuit current is also noted in the conducted experimental test by authors in their research work^58^. However, while covering the entire PV cell by implementing 100% shading, the value of ${I}_{sc}$ drops to its lowest value. These types of results are also evidently presented in the research article generated by^59^. Authors in their study concluded degradation in the ${I}_{sc}$ as one main reason for power degradation^60^. Significant changes in ${I}_{sc}$ impact the maximum generated power ${(P}_{max})$ and efficiency (*ƞ*).

#### Power output of PV module under shading condition

Two well-known performance indicators of a PV module are maximum power output (${P}_{\text{max}})$ and efficiency (*ƞ*). The experimental study is performed on single cell that represent spot shading. The results show noticeable power losses as the shading factor is increased. As the cell area is 20% shaded, a power loss of 11.6% is recorded. However, further shading 40% of the PV cell causes a rapid drop in power of 30.4%. The large variance is recorded during 40% shading, where the module power ranges from 364.822 to 253.5546 W. After shading 60% and 80% shading, the power loss stabilizes to approximately 36.18%. This is probably because, once the shaded cell is forced to function at negative voltages, the power response becomes more sensitive in order to minimize variances in cell reverse characteristics. While shading, significant changes are observed in ${I}_{sc}$, leading to a significant reduction in ${P}_{max}$ and efficiency. The experimental results of different shading scenarios are given in Table 3.

**Table 3:** Performance parameters under different conditions.

Shading percentage	Irradiation (W/m^2^)	${\text{P}}_{\text{max}}$(W)	Efficiency (ɳ) (%)	Fill factor (F.F.) (%)
No shading	1000	364.823	18.55	76.14
20% cover	1000	322.317	16.42	70.24
40% cover	1000	253.555	12.89	55.20
60% cover	1000	232.474	11.84	50.64
80% cover	1000	232.825	11.83	50.58
100% cover	1000	231.884	11.79	50.70

The data clearly reflect that shading single cell tends to reduce the overall power out, efficiency, fill factor of a module. The results corelates with the experiment conducted by Sathyanarayan et al. employing non-uniform shading and results concluded that efficiency and fill factor drastically decreased when a large cell area was shaded^61^. Shaded cells can act as resistive loads, converting the absorbed sunlight into heat instead of electricity. This localized increase in heat can create hotspots^35,62^. At the shading profiles of 40% to 60%, the maximum temperature of 30.6 °C is recorded in the shaded cell. However, upon further shading, the temperature of the shaded cell starts reducing and reaches 30.3 °C. At full coverage, the temperature elevation nearly disappeared. As observed from the experiment, the rise in temperature of the shaded cell has the potential to develop hotspots further when exposed to fielded conditions. It is essential to consider that elevated temperatures depend on the leakage current, and power is dissipated in some regions of the cell. During indoor testing, the shaded cell recorded a high temperature of 30.6 °C, while the other unshaded cell did not exhibit a significant increase in temperature. The impact of different shading profiles is observed by analysing the electrical parameters and curves and elevated temperature recorded by the data acquisition system available at the laboratory.

### Experimental analysis of outdoor testing

Apart from power loss, shading can have multiple consequences, and one of the most destructive consequences can be the development of hotspots. The hotspot temperature and its dwell time vary with the respective cells and conditions.

**Fig. 11:**
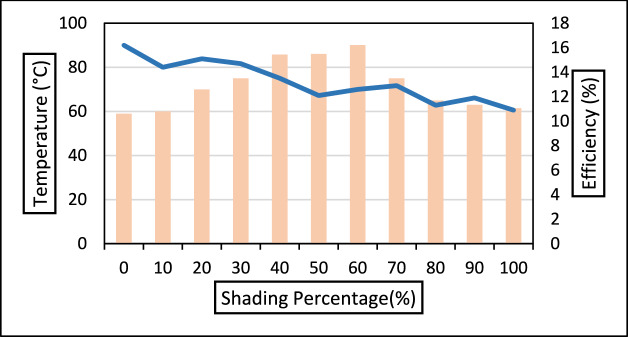
Values of maximum efficiency and recorded hotspot temperature of the shaded cell for a particular shading percentage.

Since the temperature of the hotspot is a function of irradiance, it can fluctuate during the day^34^ Also, the literature states that the amount of power dissipation at the hotspot can vary depending on the defective or mismatched cell^63^. During outdoor testing conditions, the PV module is mounted at the outdoor setup for a month and made to work in real conditions to replicate the field-installed solar modules. Using the arrangements described in Fig. 11, each shade profile is tested over one day. The readings are noted down from 08:15:00 to 17:30:00 for every minute with the help of data loggers. The module temperature during unshaded conditions is recorded to be in a range of 55–59 °C for the entire day.

When cell is stimulated with 20% shading, the temperature rises a maximum value of 70 °C. A sharp increase in shaded cell temperature is observed when the shading is increased from 40 to 60%, and the recorded values are 85.5 °C and 90.1 °C, respectively while the indoor experiments resulted maximum temperature of 30.6 °C. However, when the module was operating outdoors, it was anticipated that the shaded cell temperature would rise more under shading scenarios^64,65^. Not much variation is noted in the module temperature. During the entire day the temperature of the module under single shaded condition ranged from 59 to 62 °C during the entire experimental process and did not very much. However, in the last two shading conditions, 80–100% of a shaded cell temperature drops and is observed in the range of 65–63 °C. This occurs due to a sudden drop in photo-generated current. It is very clear from the experiment that the shading percentage from 40 to 60% is a probable partial shading condition when exhibited on a single PV, will cause maximum damage to the PV cell. Under this condition, a hotspot may develop in the shaded cell with an expected hike in temperature. This could be explained as the photocurrent declines due to shading, the cell that is shaded undergoes reverse bias causing difference in operating points of shaded and other series connected cell. This reverse characteristic of shaded cell is accountable for reverse bias process of the partially shaded cell. For conventional monocrystalline silicon cell avalanche breakdown is one of the crucial mechanism responsible for junction and this occurs at the weakest location in the cell area. The leakage current distribution might not be uniform under reverse bias condition, and one of them could lead to hotspot. Hence it a material property and the distribution of leakage current distribution that would define the rise of temperature^66^. Bressan et al. similar to this paper work explained that partial shading condition is more critical than full shading. To combat this situation they proposed real time shading fault detection that could prevent hotspotting^67^. Figure 11 represents the maximum temperature and maximum efficiency of each shading profile. The maximum temperature of the shaded part in an outdoor setup is different from that in an indoor setting. This difference is because the ambient temperature and irradiations are constant in indoor setups, while in outdoor setups, the PV module experiences a variable ambient temperature and solar radiation. Hence, the generated output is the combined effect of the persistent environmental conditions.

The current flowing through the entire string drops when even a single solar cell is shaded. As a result, each PV cell in the string will subsequently operate at the current determined by the shaded cell. The conduction of diode depends on the amount of shading and the I–V curve shape of the shaded cell. The influence on string, however, is not covered by the present experimental analysis and may be included in future research.

**Fig. 12:**
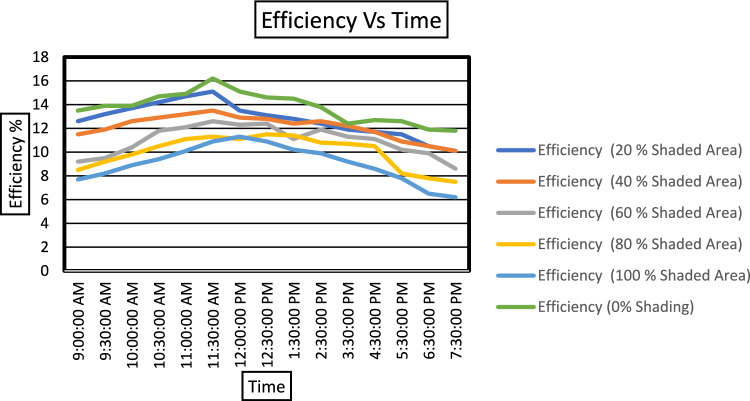
Variation in module efficiency for a full day under different shading profiles.

Figure 11 shows the value of maximum efficiency and value of maximum hotspot temperature under particular shading percentage. The temperature of the shaded cell increases till the cell area is shaded 60% of the cell. Beyond that, the temperature of the shaded cell drops. Figure 12 clearly compares the recorded efficiencies of the PV module for the entire day under respective shading profiles. The resultant efficiency of the module was obtained with a combined impact of variable solar irradiance and ambient temperature and temperature of the cell. Low irradiance and high ambient temperature lead to lower efficiency. In our experiment, the third factor, that is hotspot due to shading, is considered along with the solar irradiation and ambient temperature.

As shown in Fig. 12, the efficiency is low when the sun rises; however, as the solar radiation becomes stronger with time, the efficiency increases. However, an increase in ambient temperature along with solar radiation causes module efficiency to drop. It is observed that under the hot and dry outdoor conditions, the maximum drop in the efficiency during the experiments is recorded mainly around noon till 13:30:30, when solar radiation is not varying much and the average ambient temperature is at its peak. It is observed that when the PV module is not shaded, the module performs well with a maximum efficiency (%) of 16.25. The temperature of the cell to be tested for shading is of the same temperature as that of the PV module. The maximum efficiency during the 20% shading is generated at 11:30:00 with a solar irradiance of 960 W/m^2^. Likewise, maximum efficiency during 40%, 60%, 80%, and 100% shading is attained at 970 W/m^2^, 990 W/m^2^, 950 W/m^2^, and 940 W/m^2^, respectively. Table 4 give the values of the Maximum hotspot temperature, Maximum efficiency, maximum ambient temperature, maximum module temperature profiles during the days when the respective shading experiment is done.

**Table 4:** Temperature and Efficiency profile under shaded condition of Commercial Monocrystalline PV Module.

Shading %	Maximum cell temperature of the shaded cell (°C)	Maximum efficiency (%)	Maximum ambient temperature recorded (°C)	Maximum module temperature (°C)
0	59	16.2	32	50
20	70	15.1	32	60
40	85.8	13.5	34	61
60	90.1	12.6	35	62
80	65	11.3	34	65
100	63	11.9	34	62

The results recorded from the outdoor experiment under different shading profiles are synthesized in Table 5. The output power also declines when the shading percentage is increased, similarly as reported in the papers^68–70^. It can be noticed from the results in Table 5, that the generated output current is declining as the shading profile varies progressively from 0 to 100%. The maximum power generation (${P}_{max}$) decreased to critical during the 40–60% shading range (${P}_{max}$= 210.5 Watt and ${P}_{max}=$ 201.89 Watt, respectively).

**Table 5:** Outdoor shading scenario and power responses of single shaded PV cell.

PV module technology	Shading percentage on single cell shading					
Monocrystalline PV module	0%	20%	40%	60%	80%	100%
${I}_{mp}$[A]	8. 67	7.79	6.25	6.20	6.32	6.89
${V}_{mp}$[V]	40.15	37.98	33.68	32.50	32.56	34.56
${P}_{max}$[W]	348.10	295.86	210.5	201.89	202.78	237.08

Similar observations are recorded in an experimental investigation wherein single cell shading causes hotspot to develop and temperature of the shaded cell got elevated to 70.1 °C in case of single string configuration. However, in series–parallel connection the hotpot temperature ranged in 64.2 °C and 70.7 °C^71^. Authors Ray et al. in their work compared the performance of mono and polycrystalline solar cell. The research covers new geographical locations, a bigger data sets and a unique climatic condition offering new insights to the behaviour of solar cells. Their research work revealed dual axis tracking system offered more energy output than fixed orientation system. The methodology and finding meticulously resulted monocrystalline cell performed better in terms of injecting more energy into the grid and pursue higher performance in all the orientations^42^. Conversely Benghanem et al. conducted a similar comparative research study in hot and arid region geographical area characterised by very high solar radiation and temperature. The power losses recorded for monocrystalline modules were more than polycrystalline modules in case of high solar radiation greater than 500 $\text{w}/{\text{m}}^{2}$^72^. It is observed in several studies that diode did not conduct during single cell shading that caused shaded cell to act as a load. The temperature of the cell was observed to increase very quickly^71^. Similar observations were seen in another recent work that performed fault diagnosis on utility- scale PV projects^73^.

To authenticate the effectiveness of our experimental process under partial shading condition, the paper presents statistical results of power output, efficiency, hotspot temperature. The results presented in this work is in good agreement with the work done by Ghosh et al. and Ahan et al. Similar testing procedure on single cell shading but with different array configuration was adopted by authors Ghosh et al. The observed results showed increase in temperature up to 80.7 °C in 3 × 1 string under different partial shading condition^74^. The work presented in this paper is closely parallel to the work done by S.Ahsan et al. where similar testing methodology in conventional crystalline silicon modules. The hotspot temperature of 86.6 °C is recorded under partial shading condition^75^. It is evident that the results correlates with the finding available in the literature however this paper strictly focusses on single cell shading on new commercial high efficiency designs like PERC monocrystalline modules. A consolidate table representing the comparative values of outdoor and indoor experiments are presented in Table 6.

**Table 6:** Comparative values of indoor and outdoor experiments.

Monocrystalline PERC PV Module	${P}_{max}$		Power loss (% )		Efficiency (ɳ)	
Shading percentage	Indoor	Outdoor	Indoor	Outdoor	Indoor	Outdoor
20	322.317	295.86	11.6	15.00	16.42	15.1
40	253.555	210.5	30.4	39.52	12.89	13.5
60	232.474	201.89	36.27	42.00	11.84	12.6
80	232.825	202.78	36.18	41.75	11.83	11.3
100	231.884	237.08	36.43	31.89	11.79	11.9

## Conclusion

This paper presents a comprehend study on how commercial PERC monocrystalline PV module operated in outdoor climatic condition during single cell shading. The experiments are conducted in the a specifically chosen commercial monocrystalline PERC PV Module under the North Indian Climatic conditions of Rajasthan. In contrast to laboratory testing, the outdoor conditions are realistic and hence unpredictable. To understand the difference in the performance the shading experimental work is done in STC conditions under controlled conditions. For indoor setup the maximum power loss is of 36.34%, however for outdoor conditions the power loss of 42% is recorded. The outdoor study defines the critical shading scenario that could lead to temperature rise of about 91.1 °C in the shaded cell. Also, it shows that PV parameter show dependency on varying outdoor conditions. It is observed that in commercial PERC module the critical shading scenario ranges from 40 to 60% of the solar cell that causes hotspot development. The efficiency of the shaded module peaks from 11:00 a.m. to 11:30 a.m. thereafter the efficiency starts declining due to increase in temperature and solar irradiation having its maximum values. The results present that PERC monocrystalline modules installed in outdoor conditions pose greater challenges because of higher power losses and potential hotspot development due to single cell shading This article is beneficial for the researchers, designers, and investors working with solar power systems specifically for commercial PERC PV modules to have a critical assessment of PV power systems under shading faults.

## Data Availability

The datasets used and/or analyzed during the current study are available from the corresponding author upon reasonable request.

## Abbreviations

PV: Photovoltaic
MPP: Maximum power point
FF: Fill factor
PERC: Passivated emitter and rear contact
STC: Standard test condition
NOCT: Nominal operating cell temperature
MTC: Module test container
PLG: Pulse light generator
